# Prostate rotation detected from implanted markers can affect dose coverage and cannot be simply dismissed

**DOI:** 10.1120/jacmp.v14i3.4262

**Published:** 2013-05-06

**Authors:** Qingyang Shang, Lawrence J Sheplan Olsen, Kevin Stephans, Rahul Tendulkar, Ping Xia

**Affiliations:** ^1^ Department of Radiation Oncology Cleveland Clinic Cleveland OH USA

**Keywords:** prostate rotation, implanted markers, image‐guided radiotherapy, IMRT, image registration

## Abstract

With implanted markers, daily prostate displacements can be automatically detected with six degrees of freedom. The reported magnitudes of the rotations, however, are often greater than the typical range of a six‐degree treatment couch. The purpose of this study is to quantify geometric and dosimetric effects if the prostate rotations are not corrected (ROT_NC) and if they can be compensated with translational shifts (ROT_C). Forty‐three kilovoltage cone‐beam CTs (KV‐CBCT) with implanted markers from five patients were available for this retrospective study. On each KV‐CBCT, the prostate, bladder, and rectum were manually contoured by a physician. The prostate contours from the planning CT and CBCT were aligned manually to achieve the best overlaps. This contour registration served as the benchmark method for comparison with two marker registration methods: (a) using six degrees of freedom, but rotations were not corrected (ROT_NC); and (b) using three degrees of freedom while compensating rotations into the translational shifts (ROT_C). The center of mass distance (CMD) and overlap index (OI) were used to evaluate these two methods. The dosimetric effects were also analyzed by comparing the dose coverage of the prostate clinical target volume (CTV) in relation to the planning margins. According to our analysis, the detected rotations dominated in the left–right axis with systematic and random components of 4.6° and 4.1°, respectively. When the rotation angles were greater than 10°, the differences in CMD between the two registrations were greater than 5 mm in 85.7% of these fractions; when the rotation angles were greater than 6°, the differences of CMD were greater than 4 mm in 61.1% of these fractions. With 6 mm/4 mm posterior planning margins, the average difference between the dose to 99% (D99) of the prostate in CBCTs and the planning D99 of the prostate was −8.0±12.3% for the ROT_NC registration, and −3.6±9.0% for the ROT_C registration (p=0.01). When the planning margin decreased to 4 mm/2 mm posterior, the average difference in D99 of the prostate was −22.0±16.2% and −15.1±15.2% for the ROT_NC and ROT_C methods, respectively (p<0.05). In conclusion, prostate rotation cannot be simply dismissed, and the impact of the rotational errors depends on the distance between the isocenter and the centroid of implanted markers and the rotation angle.

PACS number: 87.55

## I. INTRODUCTION

During radiotherapy of patients with prostate cancer, the prostate position may vary due to the changes in the filling of the bladder and rectum.^1^, ^2^ Such variations pose a great challenge to the precision of treatment delivery. The use of image‐guided radiotherapy (IGRT) improves treatment precision considerably by correcting daily patient setup error and internal organ motion. Most target localization corrections from IGRT, however, are limited in translation only. Rotational setup error and rotational organ motion have been reported, but often dismissed clinically.

For patient positional setup errors, which are often detected by registering bony structures from the verification images with those from the planning images, most studies reported that rotational setup errors were relatively small with a standard deviation of about 1° around each axis.^3^, ^4^, ^5^, ^6^ Because of the small magnitude, some have suggested that the rotational errors can be ignored,^7^, ^8^ while others have suggested use of a robotic treatment couch to correct for this magnitude of rotation.^(9)^

For intertreatment organ motion of the prostate, several studies showed that the prostate organ rotation could be greater than setup error and might have an important dosimetric impact. ^3^, ^10^, ^11^, ^12^, ^13^, ^14^, ^15^, ^16^, ^17^, ^18^, ^19^ Large prostate rotations are often reported by registering the implanted markers between the verification and planning images. Rotations around left–right (LR) axis were found to be dominant because of influence of the filling of the rectum. Deutschmann et al.^(18)^ found the average LR rotation was 5.3°±4.9°, with maximum at 30.7° for 31 patients. Lips et al.^(19)^ reported rotational errors with systematic error of 6.3° and random error of 4.9°, ranging from −12.1° to 9.1° for a cohort of 19 patients.

Although using implanted markers as a surrogate to localize the prostate is a well‐adopted method for daily IGRT,^20^, ^21^ large rotations reported from the implanted marker registration are beyond the maximum correction ranges for most commercially available six‐degree couches. Others may even question the accuracy of such large rotations, and how the stability of the markers and their implanted locations affect the accuracy of these detected rotations. The purpose of this study is to quantify geometric and dosimetric effects with and without compensation for rotations detected based on marker registration, rather than to determine accuracy of the rotations detected from the marker registration.

## II. MATERIALS AND METHODS

### A. Patient selection and treatment planning

Five patients, who underwent definitive external beam radiotherapy for prostate cancer, had three electromagnetic transponders implanted in the prostate for daily IGRT, using the Calypso 4D localization system (Calypso Medical, Seattle, WA). In addition, weekly or daily kilovoltage cone‐beam CTs (KV‐CBCT) were also acquired to cross‐check the Calypso system, as needed, upon the request of radiation oncologists. A total of 43 KV‐CBCTs were available for this retrospective study.

The patients were treated with two different dose schemes: 2 Gy per fraction to a total dose of 78 Gy, and 2.5 Gy per fraction to a total dose of 70 Gy. For the purpose of this study, the prescription dose for all plans was renormalized to 2 Gy per fraction to a total dose of 78 Gy without altering IMRT optimization. The CTV was the prostate and the organs at risk (OAR) were the bladder and rectum. The clinical planning margins for these patients were 6 mm/4 mm posterior. The IMRT plans were created with the Pinnacle treatment planning system (Pinnacle^3^, 8.0m–9.0, Philips Radiation Oncology System, Madison, WI), using a typical five beam arrangement with 10 MV photon beams.

### B. Quantification of the prostate displacement

On each KV‐CBCT, the prostate, rectum, and bladder were manually contoured by a physician. Subsequently, each CBCT was registered with the corresponding planning CT using four different alignment methods (a total of 172 imaging registrations) including (i) manually align the bones using three degrees of freedom (three translations only) (BoneT); (ii) automatically align the three markers using six degrees of freedom (three translations and three rotations), but rotations are not corrected (ROT_NC); (iii) manually align the three markers using three degrees of freedom, which partially compensates rotations with translational shifts (ROT_C); and (iv) manually align the prostate contour using three degrees of freedom (ContourT), which also partially compensates rotations with translational shifts. After subtracting translational shifts from the bony alignment (BoneT), patient setup error was removed from the other three alignment methods. All the reported shifts in this paper were the prostate displacements relative to the pelvic bones.

To quantify rotations of the prostate, overall mean and standard deviation of the rotational errors were determined from the measurements of all patients. Systematic and random errors were calculated according to the method published by Remeijer et al.^(5)^ The systematic error of the entire group is the standard deviation of the individual patient means corrected for the limited and different number of measurements for each patient. The random error of the entire group is the root mean square of the individual patient standard deviation, also weighted by the number of measurements for each patient.

To investigate whether the rotations of the prostate can be compensated with translational shifts, we used the contour registration method (ContourT) as a benchmark to evaluate the two marker registration methods (ROT_NC and ROT_C) by comparing their geometric and dosimetric indices.

### C. Marker migration and false identification

Marker migration is critical for detection of the daily prostate rotation. It can be quantified by measuring the intermarker distance variation of the implanted markers between the planning CT and the daily CBCT. Besides marker migration, other causes that might produce intermarker distance variation include organ shrinkage, organ deformation, and position localization errors of the markers. These factors can affect the accuracy of the detected rotations. To examine the stability and localization error of the implanted markers, we measured the intermarker distances of the treatment day with detected rotations greater than 10° and compared them with the intermarker distances in the planning CT. We also simulated marker migration/false identification by manually adjusting the position of one of the three markers to investigate how it affects the detected rotations. For 7 fractions with rotations greater than 10°, we moved the selected marker by 1 mm and 2 mm from its original position in axial plane and moved one slice thickness (1.5 mm) superiorly and inferiorly.

### D. Geometric analysis

**Figure 1:**
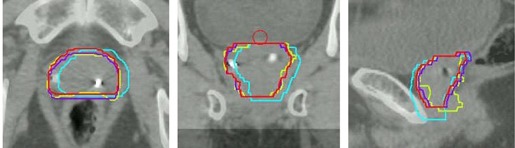
Prostate contours from different registration methods in (a) transverse, (b) coronal, and (c) sagittal views. Prostate ROT_NC is shown in blue, ProstateROT_C in red, ProstateContourT in purple, and ProstateCBCT in yellow.

To minimize potential prostate contour variations in daily CBCT, manually contoured prostate on each CBCT, denoted as ProstateCBCT, was used only for contour‐based alignment, not for geometric analysis. After each image registration, we used the transferred contours from the planning CT for geometric analysis. The transferred contours, denoted as ProstateROT_NC, ProstateROT_C, and ProstateContourT, corresponded to the ROT_NC, ROT_C, and ContourT registrations, respectively. All these contours were the CTV contours. For the ROT_NC registration, rotations were ignored for contour transferring, reflecting a certain clinical scenario in which a registration with six degrees of freedom was performed, but only translational shifts were corrected. Figure 1 is an example illustrating these prostate contours obtained from different registration methods.

#### D.1 Center of mass distance

For each of these prostate contours, a center of mass (COM) was calculated in the same CBCT frame. As a global measure of daily prostate displacement, the center‐of‐mass distance (CMD) of the prostate contours between the bony registration and each of the other three registration methods was calculated using the following equation:

(1) $$d=\sqrt{{\left(x‐x_{0}\right)}^{2}+{\left(y‐y_{0}\right)}^{2}+{\left(z‐z_{0}\right)}^{2}}$$

where *d* is the CMD, and *x, y*, and *z* are the coordinates of the COM of the transferred prostate contour after each registration method, and $x_{0},y_{0}$, and $z_{0}$ are the coordinates of the COM of the transferred prostate contour after bony registration. Using this equation, we verified the result of the contour‐based registration by calculating the relative CMD between the ProstateCBCT and the ProstateContourT.

Using the contour registration (ContourT) as a benchmark, we compared the accuracy of the two marker registration methods (ROT_NC and ROT_C) by calculating the relative CMDs between the ProstateContourT and ProstateROT_NC, and between the ProstateContourT and ProstateROT_C.

#### D.2 Overlap index

To quantitatively evaluate the geometric properties of the two marker registration methods, we defined the volume overlap index (OI) as:

(2) $$OI=\frac{\text{Prostate}\_Contour\_T\cap T\left[\text{Prostate}\_CT\right]}{\text{Prostate}\_Contour\_T}$$

where *T* is the transformation applied to the prostate contour from the planning CT (ProstateCT) by the two marker registration methods, and the operator ∩ defines the common area between the two regions of interest. Thus we have:

(3) $$OI_{NC}=\frac{\text{Prostate}\_Contour\_T\cap \text{Prostate}\_ROT\_NC}{\text{Prostate}\_Contour\_T}$$

and

(4) $$OI_{C}=\frac{\text{Prostate}\_Contour\_T\cap \text{Prostate}\_ROT\_C}{\text{Prostate}\_Contour\_T}$$

where subscript *NC* and *C* represent the ROT_NC and ROT_C methods, respectively. OI is a volumetric measure for registration accuracy, describing the geometric overlap between the “prostate of the day” and the “prostate at planning”. Since all the contours (ProstateContourT, ProstateROT_NC, and ProstateROT_C) presented in (3), (4) are contours transferred from the planning CT based on different registration methods, OI is a value ranging between 0 and 1. The higher the value, the more the overlap between the actual target volume and the planned target volume, and thus the better the registration outcome.

### E. Dosimetric analysis

For each CBCT, three verification plans were created to calculate the radiation dose of the treatment day using the same beam configuration as the original treatment plan. The treatment isocenters for these three verification plans were placed according to the three registration methods, ROT_NC, ROT_C, and ContourT, respectively. A total of 129 verification plans were created and analyzed. Since the Hounsfield Units (HU) in CBCT is inaccurate and unreliable for dose calculation, the electron density of the CBCT was overridden with 1 g/cm^3^ for voxels inside the patient external body contour, and 0 (air) for voxels outside the external contour.

The clinical planning margins for the prostate were 6 mm, except 4 mm posterior. To investigate the effect of prostate rotation with reduced planning margins, we chose not to conduct replanning for each patient using progressively reduced planning margins, which may introduce variations in initial plan quality. Instead, we created three expanded prostates (namely Prostate‐CTVs) to simulate replanning with reduced margins. These Prostate‐CTVs were created by three‐dimensionally expanding the ProstateCBCT contour with 2 mm, 4 mm, and 6 mm /4 mm posterior. We calculated the doses to 99% (D99) of these Prostate‐CTVs. For verification plans with 6 mm and 4 mm posterior planning margins, we also evaluated the dose to 5% and 50% (D5 and D50) of the bladder and rectum.

## III. RESULTS

### A. Prostate rotations

**Figure 2:**
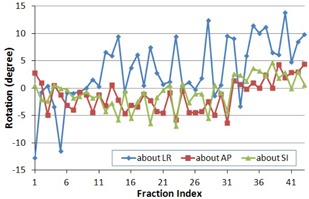
Prostate rotations about the left–right (LR), anterior–posterior (AP), and superior–inferior (SI) axes for 43 fractions.

The prostate rotations detected from the ROT_NC registration were recorded and are shown in Fig. 2 for all 43 fractions. The rotations around the anterior–posterior (AP) and superior–inferior (SI) axes were relatively small and primarily within the range of −5° and +5°, while the rotations around the left–right (LR) axis were larger, at times exceeding 10°. The overall mean and standard deviation (SD) of the rotations were 3.3°±5.8°,−1.4°±2.9°, and −0.8°±2.8°, for the LR, AP, and SI axes, respectively. The systematic SDs were 4.6°, 2.3°, and 2.1°, and the random SDs were 4.1°, 2.0°, and 2.0° for the three axes, respectively (Table 1).

**Table 1:** Prostate rotation variations (in degrees) about the left‐right (LR), anterior‐posterior (AP), and superior‐inferior (SI) axes.

	*LR*	*AP*	*SI*
Overall Mean	3.3	‐1.4	‐0.8
Overall SD	5.8	2.9	2.8
Systematic SD	4.6	2.3	2.1
Random SD	4.1	2.0	2.0

### B. Marker migration and false identification

Among 43 fractions, 7 fractions from three patients had rotations greater than 10°. The average intermarker distance for these fractions was 23.3±7.0 mm. The average absolute variation of intermarker distance was 0.8±0.6 mm. Four fractions exhibited marker migrations greater than 1 mm, with a maximum of 2.4 mm. Simulation results of marker migration/false identification showed that with 1 mm variation of the marker position, variation in rotations was less than 2°; with 2 mm variation of the marker position, the maximum variation in the rotations was 6°. Changing the marker position to its adjacent image slice (slice thickness 1.5 mm) resulted in 3° variation of the rotations.

### C. Geometric analysis

**Figure 3:**
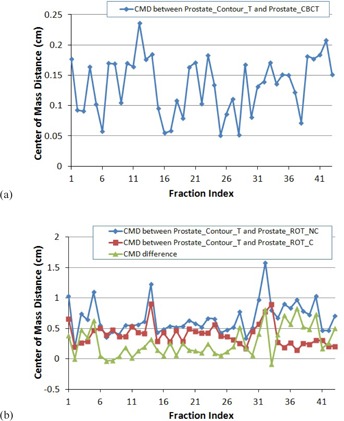
Center of mass distance (CMD): (a) between the ProstateContourT and ProstateCBCT; (b) between the Prostate ContourT and ProstateROT_NC, between the ProstateContourT and ProstateROT_C, and the difference.

The CMD between the transferred prostate contour based on contour registration and the physician drawn contour on the CBCT is shown in Fig. 3(a). This distance is a global measure of the potential prostate deformation and contouring uncertainty, which was detected to be less than 1.9 mm (1.3±0.5 mm) for 95.3% of the fractions. Thus, it is reasonable to use contour‐based registration as our benchmark for comparison of the two marker‐based registration methods.

Figure 3(b) demonstrates the CMDs between the prostates from the contour‐based registration (ContourT) and from the marker‐based registration with rotations zero out (ROT_NC), and the CMD between the prostates from the contour‐based registration and from the marker‐based translation only registration (ROT_C). Figure 3(b) also shows the CMD differences between the two marker‐based registrations. It is observed that when compared with the prostate contour from the ROT_NC registration, the prostate contour from the ROT_C registration is closer to the prostate contour from the ContourT registration, especially when the rotation is large. The mean CMDs were 6.6 mm and 3.9 mm, respectively. The difference between the two CMDs ranged from −0.9 mm to 8.3 mm when the maximum rotation (absolute value) from all three axes varied from 0.9° to 13.8°. When the rotation was greater than 10°, the difference in CMD between the prostates from the two marker‐based registrations was greater than 5 mm in 6 of 7 (85.7%) fractions. When the rotation was greater than 6°, the difference in CMD was greater than 4 mm in 11 of 18 fractions (61.1%). The statistics of the two CMDs and their differences are summarized in Table 2.

**Figure 4:**
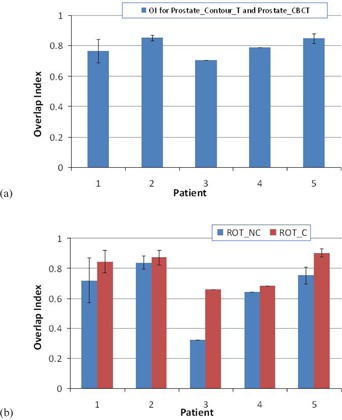
Overlap index (OI): (a) the average OI for ProstateContourT and ProstateCBCT for each patient; (b) the average OI for each patient compared between the two marker‐based registration methods. Error bar shows one standard deviation.

The average OI between the transferred prostate contour based on contour registration and the physician drawn contour on the CBCT is 0.79±0.06, as shown in Fig. 4(a). This index includes the potential prostate deformation, as well as prostate contour uncertainties in the CBCTs. We were not able to separate these two factors. However, the contouring uncertainties had a smaller impact on the CMDs. As shown in Fig. 3(a), the average CMD between the ProstateContourT and the ProstateCBCT of 1.3±0.5 mm indicated that the prostate deformation might be minimal.

Figure 4(b) compares the OI averaged over each patient for the two marker registration methods. In general, $OI_{c}$ exhibits greater value than ${OI}_{NC}$, indicating that better geometric overlap between the “prostate of the day” and the “prostate at planning” can result from the marker registration with translational correction. In three of five patients, ${OI}_{c}$ shows over 10% improvement of overlap when compared with ${OI}_{NC}$.

**Table 2:** Center of mass distance (CMD) of prostate shifts between the contour‐based and two marker‐based registrations.

	*CMD Between ROT_NC and ContourT* ^(39)^	*CMD Between ROT_C and ContourT* ^(39)^	*Difference* ^(39)^
Max.	15.8	9.1	8.3
Min.	1.9	1.4	‐0.9
Mean	6.6	3.9	2.7
SD	2.6	1.8	2.4

### D. Dosimetric analysis

**Figure 5:**
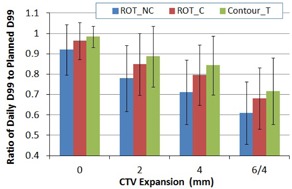
Average D99 of the Prostate‐CTV as a function of CTV expansion margins for the three registration methods. D99 is expressed as a ratio of the daily dose to the planned daily D99 of the prostate. Error bar represents one standard deviation.

Figure 5 shows the ratios of D99 of the Prostate‐CTVs from three types of verification plans to the planned D99 of the prostate with three equivalent planning margins. With 6 mm/4 mm posterior planning margins, D99 of the prostate was significantly different between the two marker registrations (p=0.01) — −3.6±9.0% difference from the planned dose for the ROT_C method, compared to −8.0±12.3% for the ROT_NC method. The dose differences (p<0.05) between the two methods increased as the planning margins decreased, as shown in Table 3. For example, with a 2 mm margin reduction (equivalent to a 2 mm expansion of the prostate in this study), the ROT_C method could improve dose coverage to the Prostate‐CTV D99 by 6.9%. From Fig. 5, the contour‐based registration achieved the best dose coverage, followed by the ROT_C method as the planning margin progressively reduced.

The dosimetric improvement of translational correction is greater when the magnitude of the rotation is large. For example, for a patient with detected rotations of 8.8°±2.9° around the LR axis, the average daily D99 of the prostate was 1.99 Gy (99.7% of the planned dose, with a daily prescription dose of 2.0 Gy) with the ROT_C method, compared to 1.68 Gy (84.4% of the planned dose) with the ROT_NC method. As the planning margin decreased to 4 mm/2 mm posterior, the average daily D99 of the Prostate‐CTV improved from 1.39 Gy from the ROT_NC method to 1.82 Gy from the ROT_C method, a 21.4% improvement by compensating rotations with translational shifts.

**Figure 6:**
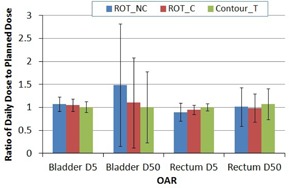
Average D5 and D50 of the bladder and rectum. Column represents mean value (normalized to the planned dose) and error bar corresponds to one standard deviation.

Figure 6 shows doses to 5% (D5) and 50% (D50) of the bladder and rectum for the two marker registration methods. D5 of the bladder and D50 of the rectum did not reach statistically significant differences (p‐values were 0.09 and 0.44, respectively) by the ROT_NC and ROT_C methods. D5 of the rectum was lower than the planned dose for both methods, and D50 of the bladder was significantly lower for the ROT_C method (p=0.004). We also noticed that the dose variation in D50 of the bladder was largely due to the large volume variation of the bladder during the treatment course. The average dose differences for OAR from all fractions between the dose of the day and the dose of the plan are listed in Table 4.

**Table 3:** Average difference in D99 of the Prostate‐CTV between the daily and planned dose.

	*CTV Expansion* ^(39)^			
*Registration Methods*	*0*	*2*	*4*	*6/4*
ROT_NC	−8.0±12.3%	−22.0±16.2%	−28.7±15.8%	−39.0±15.3%
ROT_C	−3.6±9.0%	−15.1±15.2%	−20.4±14.7%	−31.9±15.1%
ContourT	−1.6±5.2%	−11.3±15.0%	−15.6±14.4%	−28.3±16.3%
Improvement by correction	4.4%	6.9%	8.3%	7.1%

Note: The differences are expressed in percentage (%) of the planned D99 of the prostate as a function of CTV expansion margins. Negative mean value indicates less dose in daily D99 of the Prostate‐CTV when compared to the planned dose.

**Table 4:** Average dose difference for OAR between the daily and planned dose.

	*OAR Dose*			
*Registration Methods*	*Bladder D5*	*Bladder D50*	*Rectum D5*	*Rectum D50*
ROT_NC	7.2±15.9%	48.5±133.4%	−10.5±19.5%	1.2±41.7%
ROT_C	4.8±13.2%	10.1±98.4%	−4.7±10.2%	−1.3±30.5%
ContourT	0.8±11.8%	0.9±77.2%	0.2±7.9%	7.2±40.0%

Note: The differences are expressed in percentage (%) of the planned dose as the mean ± standard deviation. Negative mean value indicates better organ sparing compared with the original plan.

## IV. DISCUSSION

We investigated the magnitude and distribution of the prostate interfractional rotations and evaluated the geometric and dosimetric effects of the rotations. Precisely correcting the prostate organ rotation is clinically important but challenging, especially when rotation angles are large. Recent development of six‐degree treatment couches has enabled correction of the rotations,^9^, ^17^, ^22^, ^23^ but such treatment couches are not widely available or able to correct large magnitude of rotations. For the purpose of patient safety, the correction range for a typical six‐degree couch is about 4°–5°. Other correction strategies have been proposed, such as gantry and collimator angle adjustments to partially correct for left–right rotations,^(24)^ offline adaptive planning,^25^, ^26^, ^27^ and dynamic MLC tracking.^(28)^ However, most of these strategies are in the investigation stages and are not yet clinically practical.

During treatment for most patients with prostate cancer, patient positioning errors and prostate displacement are often not separated. In this study, we used a dual imaging registration method to separate the positioning errors and prostate displacement. Because reported rotation errors in patient positioning were small, we used the bony registration with translational shifts to determine the positional setup errors. Subtracting the translational shifts from the bone registration, the translational shifts and rotations from the marker registration were primarily from the displacement of the prostate, or artifact of the registration method.

Nevertheless, Table 5 compares our data with other reported data in the literature for both systematic and random components of prostate rotations detected with three imaging registration methods. The systematic component describes the variation of the mean displacement of the prostate, while the random component delineates the day‐to‐day position variation of the prostate. Compared to the results from marker registration, our data agree well with previous results from Dehnad et al.^(11)^ and Graf et al.^(17)^ Except for the study conducted by Owen et al.,^(16)^ prostate rotation around the LR axis was found to be the largest of the three axes. Lips et al.^(19)^ and van der Heide et al.^(20)^ found larger systematic error (6.3° and 6.8°, respectively) than others around the LR axis, and Aubry et al.^(12)^ also reported greater random error (6.1°) around the LR axis.

**Table 5:** Systematic and random components (in degrees) of prostate rotations in the literature.

		*Systematic Components*			*Random Components*		
*Authors*		*LR*	*AP*	*SI*	*LR*	*AP*	*SI*
Contour‐based Registration	Van Herk et al.^(29)^	N/A	N/A	N/A	4.0	1.3	2.1
	Stroom et al.^(10)^	3.6	0.8	1.7	3.3	0.9	1.5
	Hoogeman et al.^(13)^	5.1	1.3	2.2	3.6	1.6	2.0
Image‐based Registration	Nijkamp et al.^(25)^	2.9	0.9	1.0	3.0	1.0	1.1
Marker‐based Registration	Dehnad et al.^(11)^	4.7	2.0	2.7	3.6	1.7	1.9
	Aubry et al.^(12)^	5.6	2.2	2.4	6.1	2	2.8
	Graf et al.^(17)^	4.1	2.3	1.6	3.1	1.8	2.0
	Van der Heide et al.^(20)^	6.8	2.8	2.8	3.1	1.7	2.0
	Lips et al.^(19)^	6.3	2.0	2.8	4.9	1.0	1.4
	Owen et al.^(16)^	7.6	5.0	7.7	10.2	6.5	15.8
	Our study	4.6	2.3	2.1	4.1	2.0	2.0

The variation of the reported prostate rotations (Table 5) may stem from differences in treatment protocols, image registration approaches, and mathematical methods for error computation at each institute. Some studies matched the prostate contours in the planning CT with those in the repeated CT scans to get the rotations of the prostate relative to the pelvic bones,^3^, ^10^, ^13^ while others performed the registration based on the implanted markers.^11^, ^12^, ^16^, ^17^, ^19^, ^20^ In addition, the mathematical equations used to compute the systematic and random errors might be slightly different from one study to another. For example, Owen et al.^(16)^ computed the errors using the method described by van Herk,^(29)^ and Stroom et al.,^(10)^ and Hoogeman et al.^(13)^ utilized a similar method but corrected the systematic error for the finite number of measurements. Aubry et al.^(12)^ employed an approach by Remeijer et al.,^(5)^ not only considering the limited size of samples, but also accounting for the different number of measurements for each patient. In this study, we used the same method as that in the Remeijer study. Furthermore, the selection of rotation center has a strong effect on the resultant rotations. Owen and colleagues used the marker placed near the apex of the prostate as the pivot point for rotation computation, while for most of the other studies, rotations were measured at the centroid of the prostate contours or the centroid of the markers, depending on whether the contour‐based or marker‐based registration method is used. The Owen study reported much greater rotations (7.6°, 5.0°, and 7.7° for systematic and 10.2°, 6.5°, and 15.8° for random) than others.

In this study, the registration was performed in the treatment planning system instead of the on‐board imaging system of the linear accelerator. Daily CBCT and the planning CT are served as the primary and secondary image for fusion, respectively. The secondary image is translated and rotated to match the primary image. The resultant rotations were measured around the image volume center of the secondary image (planning CT). Rotations could also be measured at the treatment isocenter.^(30)^ However, the centroid of the markers, which in general represents the center of mass of the prostate organ, could be different from the treatment isocenter or the image center. For example, for concurrent treatment of the prostate and pelvic lymph nodes, the isocenter is usually placed outside of the prostate. Isocenter may also be adjusted away from the center of the prostate because of practical reasons. In these cases, if rotations are measured around the isocenter, or any point other than the centroid of the markers (e.g., image volume center), ignoring such rotation will introduce a translational error at the centroid of the markers, indicating prostate displacement from its supposed position. For instance, Linthout et al.^(23)^ reported that a tilt rotation of 2.5° at the foot end of the couch could lead to a vertical shift of 4.5 cm at the isocenter for a standard prostate cancer patient positioning (where the isocenter is close to the center of the prostate).

**Figure 7:**
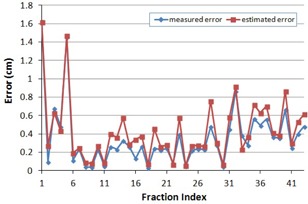
The magnitude of measured and estimated errors associated with the ROT_NC method.

The ROT_NC method obtains a six‐degree‐of‐freedom solution, but only applies the translational components of the solution for correction. In the scenario where the rotation center is different from the centroid of the markers, only the rotation center is corrected precisely with the ROT_NC method (under the assumption of no prostate deformation and other uncertainties such as marker migration). For all the other points in the image, especially for points inside the prostate which we are interested in, there will be a translational error produced by the uncorrected rotation. The magnitude of the error depends on the distance to the rotation center and the detected rotation at that point. Mathematically, the translational error at the centroid of the markers due to uncorrected rotations can be estimated roughly by Eq. (5) according to the law of cosines in Euclidean geometry:

(5) $$error=D\sqrt{2\left(1‐\text{cos}\theta \right)}$$

where *D* is the distance between the centroid of the markers and the rotation center, and θ is the rotation angle about the LR axis (assuming rotations about the AP and SI axes are small enough to be neglected). Figure 7 shows the magnitude of the measured and estimated error of the ROT_NC method at the centroid of the markers from its true position where rotations are corrected precisely. Except for a few fractions where the assumption of small rotations about the other two axes was not satisfied, the measured errors agreed well with the estimated ones for the majority of fractions. A greater distance D and/or larger rotation angle θ creates larger error. For example, for a patient with D=7.3 cm and θ=4.9°, the measured translational error associated with the ROT_NC method was 6.8 mm; for another patient with D=3.5 cm and θ=4.5°, the error was 2.2 mm. For the same patient with D=3.5 cm, the error was 4.0 mm when the detected rotation was 9.4°, compared to 0.5 mm error when the rotation was less than 1°.

On the other hand, the ROT_C method uses only three degrees of freedom for correction. The resultant translational solution contains two separable components. The first component corrects the initial translational error (three translations from the six‐degree‐of‐freedom solution) to the rotation center; and the second component compensates for translations caused by the rotation for points in the prostate by matching the three markers. Therefore, the centroid of the three implanted markers is precisely corrected (theoretically, with the same assumption as above). For other points inside the prostate, the second translational component only partially corrects for rotations. Thus, the residual translational errors of the prostate from the ROT_C method are smaller than those from the ROT_NC method. Using contour‐based registration as our benchmark, the displacement of the prostate from its supposed position after correction by the two marker‐based registration methods was quantified by the center of mass distance and overlap index (3, 4). In the scenario where the rotation center is the centroid of the prostate, the ROT_NC method will produce the same results as that of the ROT_C method. To avoid substantial shifts of the prostate centroid caused by the ROT_NC method, placing the isocenter close to the centroid of the implanted markers is recommended. Otherwise, using the ROT_C method is recommended.

The detected rotations from marker registration may depend on spatial relationship of the implanted markers, marker stability within the prostate, and the accuracy of the marker identification. It is speculated that when markers are implanted close to each other, large organ rotations may be falsely rendered. Migration or localization errors of implanted markers could also lead to falsely rendered rotations. Although several studies reported that the average marker movement was very small, on the order of 1 mm,^11^, ^31^, ^32^, ^33^, ^34^ there was evidence of relatively large intermarker distance variations for individual patients. McNair et al.^(35)^ found marker migration of more than 2 mm in 10% (3 of 30) patients; Deutschmann et al.^(18)^ observed 24 of 342 patients had intraprostatic migrations of one of four markers greater than 3 mm, and 10 greater than 4 mm; while Kupelian et al.^(34)^ reported that in 47 of 56 patients (84%), the maximum intermarker distance variation was at least 2 mm, and the percentage for 3, 4, 5 mm variation were 41%, 18%, and 9%, respectively. They also reported that the maximum standard deviation of the intermarker distance was 4.2 mm and the maximum observed variation was 10.2 mm. The infrequent yet potential marker migrations may have more profound effect on the resulted rotations than translations.^(18)^

By examining the intermarker distance variation of fractions with detected rotations greater than 10°, we observed the maximum variation of the Calypso transponders exceeded 2 mm in two patients. Studies have shown that the migration of Calypso transponders is within the similar range of gold markers routinely implanted for prostate treatment.^(36)^ When large intermarker distance variations (up to a few millimeters) occurred, either substantial marker migration or significant organ deformation was indicated.^31^, ^34^ In these cases, the rotations detected by marker registration may not truly represent the actual organ displacement. Our simulation experiment reported up to 6° of rotation variation resulted from 2 mm marker migration/false identification, indicating that a small migration of the implanted markers or a small localization error in marker positions may result in a large degree of rotation. Such uncertainties in rotation detection will further hamper the accuracy of rotation correction. On the other hand, marker migrations and false identification will have less influence on the accuracy of the translational shifts. Using translational shifts to compensate for rotational error of the prostate is a safe strategy which will not be affected substantially by the potential marker migration or localization errors of the marker positions.

Mutanga et al.^(37)^ found the benefit of rotation corrections was insignificant for systematic error around the LR axis of 4.3° and random error of 4.5° for patients receiving treatment to the prostate only. With 3 mm uniform planning margin, they reported the average increase in population $D_{\min}$ with rotation corrections was only 0.3±0.8 Gy. However, Lips et al.^(19)^ observed patients with large rotations had considerable dose reduction in the CTV in a study with planning margins of 2, 4, 6, and 8 mm. It was concluded that without correcting the rotation errors, online translational correction produced little improvement compared with off‐line verification for complex prostate IMRT plans. A study by Li et al.^(30)^ also shown that the dosimetric impact of prostate rotation was more significant than the impact of translational shifts in intrafractional motion. They concluded that the treatment margin can be reduced substantially if the residual rotational errors can be managed within 1° in any direction. In our study, we found that correcting rotation errors becomes more important when a smaller planning margin is used to reduce normal tissue toxicity, as well as for patients with greater distances between the centroid of the markers and the rotation center, and patients with large rotation angles. For example, for patients with distances 7.3 cm and 3.5 cm, the daily D99 of the prostate was 1.82 Gy (90.9% of the planned dose) and 1.91 Gy (95.3% of the planned dose), respectively, for the ROT_NC method when the rotation was about 4° for both cases. As the planning margin decreased from 6 mm/4 mm to 4 mm/2 mm, and further to 2 mm/0 mm posterior, the improvement of D99 of the prostate with translational correction increased from 4.4% to 6.9%, and further to 8.3% (Table 3). Such improvement in D99 is even greater for patients exhibiting large rotations. For example, for a selected patient with rotations of 8.8°±2.9°,2.0°±1.7°, and 2.3°±1.5° around the LR, AP, and SI axes, for planning margins of 6 mm/4 mm, 4 mm/2 mm, and 2 mm/0 mm posterior, the translational correction method improved D99 of the prostate by 15.3%, 21.4%, and 25.9%, respectively.

It should be noted that, in this study, we only investigated the rotations from the prostate motion alone. We did not specifically examine the movement of the seminal vesicles because fiducial markers were not routinely implanted in the seminal vesicles to monitor the organ motion. However, the dosimetric impact of rotation errors on seminal vesicles is worthy of further investigation, since it is known that the seminal vesicles move even more than the prostate.^(38)^

## V. CONCLUSIONS

This study indicates that the rotation of the prostate resulting from marker registration may be substantial. Without correcting the rotation error, inadequate dosimetric coverage to the prostate may result, especially when the rotations are large. Purposely placing the isocenter, which is presumed to be the rotation center of the image registration method, close to the centroid of the implanted markers can minimize the potential rotation error when an automatic marker registration method is applied without a six‐degree couch. Otherwise, manually registering the implanted markers with translational shifts, which partially compensates rotations, is recommended, especially when the magnitude of the rotation is large.

## ACKNOWLEDGMENTS

This research is supported in part by the United States Army Medical Research and Material Command (USAMRMC, PC073349).

## References

[1] Langen KM and Jones DT . Organ motion and its management. Int J Radiat Oncol Biol Phys. 2001;50(1):265–78. 11316572 10.1016/s0360-3016(01)01453-5

[2] Byrne TE . A review of prostate motion with considerations for the treatment of prostate cancer. Med Dosim. 2005;30(3):155–61. 16112467 10.1016/j.meddos.2005.03.005

[3] van Herk M , Bruce A , Kroes AP , Shouman T , Touw A , Lebesque JV . Quantification of organ motion during conformal radiotherapy of the prostate by three dimensional image registration. Int J Radiat Oncol Biol Phys. 1995;33(5):1311–20. 7493856 10.1016/0360-3016(95)00116-6

[4] Hanley J , Lumley MA , Mageras GS , et al. Measurement of patient positioning errors in three‐dimensional conformal radiotherapy of the prostate. Int J Radiat Oncol Biol Phys. 1997;37(2):435–44. 9069319 10.1016/s0360-3016(96)00526-3

[5] Remeijer P , Geerlof E , Ploeger L , Gilhuijs K , van Herk M , Lebesque J V . 3‐D portal image analysis in clinical practice: an evaluation of 2‐D and 3‐D analysis techniques as applied to 30 prostate cancer patients. Int J Radiat Oncol Biol Phys. 2000;46(5):1281–90. 10725642 10.1016/s0360-3016(99)00468-x

[6] Guckenberger M , Meyer J , Vordermark D , Baier K , Wilbert J , Flentje M . Magnitude and clinical relevance of translational and rotational patient setup errors: a cone‐beam CT study. Int J Radiat Oncol Biol Phys. 2006;65(3):934–42. 16751076 10.1016/j.ijrobp.2006.02.019

[7] Fu W , Yang Y , Li X , Heron DE , Huq MS , Yue NJ . Dosimetric effects of patient rotational setup errors on prostate IMRT treatments. Phys Med Biol. 2006;51(20):5321–31. 17019041 10.1088/0031-9155/51/20/016

[8] Cranmer‐Sargison G . A treatment planning investigation into the dosimetric effects of systematic prostate patient rotational set‐up errors. Med Dosim. 2008;33(3):199–205. 18674684 10.1016/j.meddos.2007.06.005

[9] Soete G , Verellen D , Tournel K , Storme G . Setup accuracy of stereoscopic X‐ray positioning with automated correction for rotational errors in patients treated with conformal arc radiotherapy for prostate cancer. Radiother Oncol. 2006;80(3):371–73. 16914219 10.1016/j.radonc.2006.07.001

[10] Stroom JC , Koper PC , Korevaar GA , et al. Internal organ motion in prostate cancer patients treated in prone and supine treatment position. Radiother Oncol. 1999;51(3):237–48. 10435819 10.1016/s0167-8140(99)00061-4

[11] Dehnad H , Nederveen AJ , van der Heide UA , van Moorselaar RJA , Hofman P , Lagendijk JJW . Clinical feasibility study for the use of implanted gold seeds in the prostate as reliable positioning markers during megavoltage irradiation. Radiother Oncol. 2003;67(3):295–302. 12865177 10.1016/s0167-8140(03)00078-1

[12] Aubry JF , Beaulieu L , Girouard LM , et al. Measurements of intrafraction motion and interfraction and intrafraction rotation of prostate by three‐dimensional analysis of daily portal imaging with radiopaque markers. Int J Radiat Oncol Biol Phys. 2004;60(1):30–39. 15337537 10.1016/j.ijrobp.2004.02.045

[13] Hoogeman MS , van Herk M , de Bois J , Lebesque JV . Strategies to reduce the systematic error due to tumor and rectum motion in radiotherapy of prostate cancer. Radiother Oncol. 2005;74(2):177–85. 15734206 10.1016/j.radonc.2004.12.010

[14] Wu QW , Ivaldi G , Liang J , Lockman D , Yan D , Martinez A . Geometric and dosimetric evaluations of an online image‐guidance strategy for 3D‐CRT of prostate cancer. Int J Radiat Oncol Biol Phys. 2006;64(5):1596–609. 16580509 10.1016/j.ijrobp.2005.12.029

[15] Boda‐Heggemann J , Kohler F , Wertz H , et al. Fiducial‐based quantification of prostate tilt using cone beam computer tomography (CBCT). Radiother Oncol. 2007;85(2):247–50. 17935807 10.1016/j.radonc.2007.09.008

[16] Owen R , Kron T , Foroudi F , Milner A , Cox J , Duchesne G . Interfraction prostate rotation determined from in‐room computerized tomography images. Med Dosim. 2011;36(2):188–94. 21540013 10.1016/j.meddos.2010.03.002

[17] Graf R , Boehmer D , Budach V , Wust P . Interfraction rotation of the prostate as evaluated by kilovoltage X‐ray fiducial marker imaging in intensity‐modulated radiotherapy of localized prostate cancer. Med Dosim. 2012;37(4):396–400. 22534137 10.1016/j.meddos.2012.02.006

[18] Deutschmann H , Kametriser G , Steininger P , et al. First clinical release of an online, adaptive, aperture‐based image‐guided radiotherapy strategy in intensity‐modulated radiotherapy to correct for inter‐ and intrafractional rotations of the prostate. Int J Radiat Oncol Biol Phys. 2012;83(5):1624–32. 22209149 10.1016/j.ijrobp.2011.10.009

[19] Lips IM , van der Heide UA , Kotte AN , van Vulpen M , Bel A . Effect of translational and rotational errors on complex dose distributions with off‐line and on‐line position verification. Int J Radiat Oncol Biol Phys. 2009;74(5):1600–08. 19473778 10.1016/j.ijrobp.2009.02.056

[20] van der Heide UA , Kotte AN , Dehnad H , Hofman P , Lagenijk JJ , van Vulpen M . Analysis of fiducial marker‐based position verification in the external beam radiotherapy of patients with prostate cancer. Radiother Oncol. 2007;82(1):38–45. 17141903 10.1016/j.radonc.2006.11.002

[21] Khosa R , Nangia S , Chufal KS , Ghosh D , Kaul R , Sharma L . Daily online localization using implanted fiducial markers and its impact on planning target volume for carcinoma prostate. J Cancer Res Ther. 2010;6(2):172–78. 20622364 10.4103/0973-1482.65244

[22] van Herten YR , van de Kamer JB , van Wieringen N , Pieters BR , Bel A . Dosimetric evaluation of prostate rotations and their correction by couch rotations. Radiother Oncol. 2008;88(1):156–62. 18439697 10.1016/j.radonc.2008.03.016

[23] Linthout N , Verellen D , Tournel K , Reynders T , Duchateau M , Storme G . Assessment of secondary patient motion induced by automated couch movement during on‐line 6 dimensional repositioning in prostate cancer treatment. Radiother Oncol. 2007;83(2):168–74. 17499870 10.1016/j.radonc.2007.04.015

[24] Rijkhorst EJ , Van Herk M , Lebesque J V , Sonke JJ . Strategy for online correction of rotational organ motion for intensity‐modulated radiotherapy of prostate cancer. Int J Radiat Oncol Biol Phys. 2007;69(5):1608–17. 17919845 10.1016/j.ijrobp.2007.08.042

[25] Nijkamp J , Pos FJ , Nuver TT , et al. Adaptive radiotherapy for prostate cancer using kilovoltage cone‐beam computed tomography: first clinical results. Int J Radiat Oncol Biol Phys. 2008;70(1):75–82. 17869445 10.1016/j.ijrobp.2007.05.046

[26] Lei Y and Wu QW . A hybrid strategy of offline adaptive planning and online image guidance for prostate cancer radiotherapy. Phys Med Biol. 2010;55(8):2221–34. 20354283 10.1088/0031-9155/55/8/007 PMC3042853

[27] Liu H and Wu QW . Dosimetric and geometric evaluation of a hybrid strategy of offline adaptive planning and online image guidance for prostate cancer radiotherapy. Phys Med Biol. 2011;56(15):5045–62. 21772083 10.1088/0031-9155/56/15/024 PMC3145215

[28] Wu J , Ruan D , Cho B , et al. Electromagnetic detection and real‐time DMLC adaptation to target rotation during radiotherapy. Int J Radiat Oncol Biol Phys. 2012;82(3):e545–53. 22014957 10.1016/j.ijrobp.2011.06.1958

[29] van Herk M . Errors and margins in radiotherapy. Semin Radiat Oncol. 2004;14(1):52–64. 14752733 10.1053/j.semradonc.2003.10.003

[30] Li JS , Jin L , Pollack A , et al. Gains from real‐time tracking of prostate motion during external beam radiation therapy. Int J Radiat Oncol Biol Phys. 2009;75(5):1613–20. 19836164 10.1016/j.ijrobp.2009.05.022

[31] Poggi MM , Gant DA , Sewchand W , Warlick WB . Marker seed migration in prostate localization. Int J Radiat Oncol Biol Phys. 2003;56(5):1248–51. 12873668 10.1016/s0360-3016(03)00328-6

[32] Litzenberg D , Dawson LA , Sandler H , et al. Daily prostate targeting using implanted radiopaque markers. Int J Radiat Oncol Biol Phys. 2002;52(3):699–703. 11849792 10.1016/s0360-3016(01)02654-2

[33] Pouliot J , Aubin M , Langen KM , et al. (Non)‐migration of radiopaque markers used for on‐line localization of the prostate with an electronic portal imaging device. Int J Radiat Oncol Biol Phys. 2003;56(3):862–66. 12788196 10.1016/s0360-3016(03)00267-0

[34] Kupelian PA , Willoughby TR , Meeks SL , et al. Intraprostatic fiducials for localization of the prostate gland: monitoring intermarker distances during radiation therapy to test for marker stability. Int J Radiat Oncol Biol Phys. 2005;62(5):1291–96. 16029784 10.1016/j.ijrobp.2005.01.005

[35] McNair HA , Hansen VN , Parker CC , et al. A comparison of the use of bony anatomy and internal markers for offline verification and an evaluation of the potential benefit of online and offline verification protocols for prostate radiotherapy. Int J Radiat Oncol Biol Phys. 2008;71(1):41–50. 17996391 10.1016/j.ijrobp.2007.09.002

[36] Willoughby TR , Kupelian PA , Pouliot J , et al. Target localization and real‐time tracking using the Calypso 4D localization system in patients with localized prostate cancer. Int J Radiat Oncol Biol Phys. 2006;65(2):528–34. 16690435 10.1016/j.ijrobp.2006.01.050

[37] Mutanga TF , de Boer HC , van der Wielen GJ , Hoogeman MS , Incrocci L , Heijmen BJ . Margin evaluation in the presence of deformation, rotation, and translation in prostate and entire seminal vesicle irradiation with daily marker‐based setup corrections. Int J Radiat Oncol Biol Phys. 2011;81(4):1160–67. 21035957 10.1016/j.ijrobp.2010.09.013

[38] Beard CJ , Kijewski P , Bussiere M , et al. Analysis of prostate and seminal vesicle motion: implications for treatment planning. Int J Radiat Oncol Biol Phys. 1996;34(2):451–58. 8567348 10.1016/0360-3016(95)02081-0

